# Preliminary validation of the Klenico diagnostic software self-report module through comparison with the diagnostic gold standard in an outpatient routine clinical sample

**DOI:** 10.1080/21642850.2023.2244576

**Published:** 2023-08-30

**Authors:** Stefan Reutimann, David Milanovic, Marco D. Gulewitsch, Mareike Augsburger

**Affiliations:** a Klenico Health AG, University of Zurich Startup, Zürich, Switzerland; b Department of Psychology, Faculty of Science, University of Tübingen, Tübingen, Germany

**Keywords:** Klenico, clinical assessment, validation, gold standard, mental disorders

## Abstract

**Background:**

Inaccuracy in current diagnostic procedures for mental disorders can lead to misdiagnosis and increase the burden on the healthcare system. Therefore, Klenico, a diagnostic software designed to support comprehensive and efficient clinical diagnostic procedures that is easy to apply in everyday clinical practice, was developed. This study aimed to take the first step toward validating the Klenico self-report module.

**Methods:**

Data of 115 patients from a German psychotherapeutic outpatient clinic were included in this study. Criterion validity was tested by comparing Klenico with the diagnoses based on the structured clinical interview for DSM-IV (SCID). Construct validity was investigated by comparing Klenico with commonly used self-reporting questionnaires.

**Results:**

The results showed that most of the Klenico disorder domains were able to differentiate between corresponding diagnoses and other diagnoses, confirming criterion validity. Construct validity was demonstrated by high correlations with the compared convergent questionnaire scales and non-significant or low correlations with most of the divergent scales.

**Conclusions:**

These preliminary results demonstrate the psychometric properties of the Klenico self-report module and imply that the Klenico system has high potential to improve the accuracy of diagnostic procedures in everyday clinical practice.

## Introduction

The high prevalence of mental disorders in Europe (Steel et al., 2014) poses a growing burden and challenge to its healthcare systems and society (Grandes et al., 2011). Accurate diagnosis is essential to guide patients to appropriate and efficient treatment (Bukh et al., 2013). State-of-the-art clinical diagnostics include an initial interview, which serves as a general clarification and can be supplemented with screening questionnaires. Subsequently, clinicians diagnosis patients using standardized tools; in particular (semi-)structured interviews, which are considered the gold standard due to their reliability and validity (Nakash et al., 2015).

However, in clinical practice, professionals frequently rely on unstructured free assessments (Bruchmüller et al., 2011). While these are perceived as less time-consuming, they often lack validity and reliability (Miller et al., 2001). Admittedly, even with extensive experience it is impossible to be aware of all the diagnostic criteria for disorders (Rief & Stenzel, 2012). Moreover, disorder-specific questionnaires designed to assess severity are often used as the sole tool for diagnostic purposes. These selective or insufficiently standardized procedures pose the dual risk of incorrect diagnoses and disregarding comorbidities (Newson et al., 2020), which can ultimately result in longer non-indication-based therapies and unnecessary health care expenses (Carvalho & McIntyre, 2017).

To address these challenges, Klenico was developed as a low-threshold diagnostic tool that covers a wide range of mental disorders, implements state-of-the-art diagnostic procedures, and is simple to implement in clinical practice.

### Klenico system

Klenico is an online-based diagnostic software that includes most mental disorders outlined in both the Diagnostic and Statistical Manual of Mental Disorders, Fifth Edition (DSM-5) and the International Classification of Diseases Version 10 (ICD-10). To date, the following disorder domains have been implemented: anxiety disorders (agoraphobia, generalized anxiety disorder (GAD), panic disorder, social phobias, specific phobias); disorders of adult personality and behavior; eating disorders (anorexia nervosa, bulimia nervosa); mental and behavioral disorders due to psychoactive substance use; mood (affective) disorders (depressive disorders, mania); obsessive-compulsive disorder (OCD); reaction to severe stress and adjustment disorders; and psychotic disorders (schizophrenia, schizotypal, and delusional disorders). Aspects of autism spectrum disorder, attention deficit hyperactivity disorder (ADHD), and dementia have also been included. The system further contains items related to severe symptoms (i.e. self-harm, potential harm to others, and suicidal ideation).

Klenico was developed in collaboration with clinical experts to ensure high content validity. Items were formulated based on the symptom criteria of the DSM-5 and ICD-10 and cross-checked with the experts in an iterative fashion. For further details on the development of the Klenico system, see Lustig (2020).

**Figure 1.**
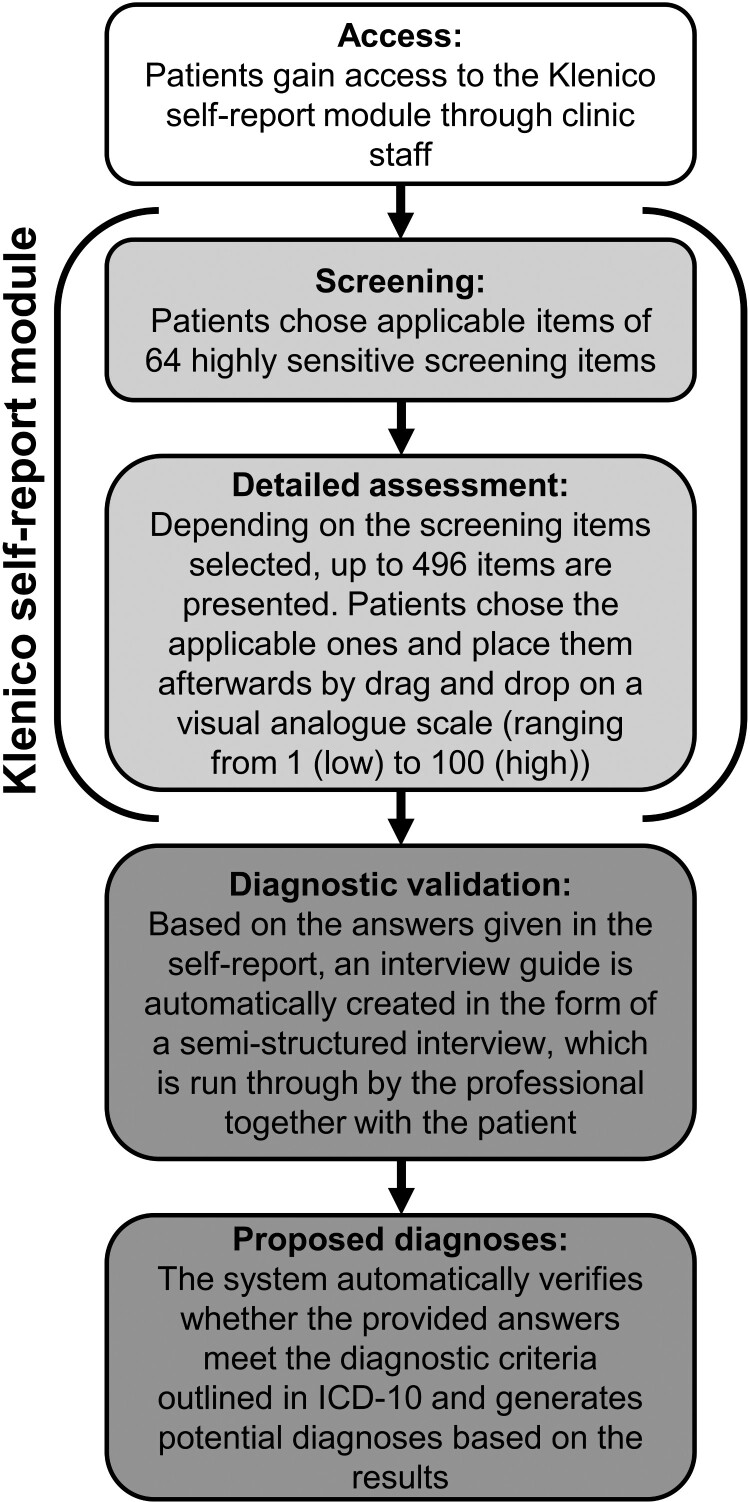
Different steps of the Klenico system including self-report module and diagnostic validation module. Off note, only the self-report module was assessed in the current analysis.

Klenico consists of a self-report module which is answered independently by the patients on laptop, mobile phone or tablet and a subsequent clinical validation module in the form of a semi-structured interview conducted by clinicians together with the patients. In the process of the diagnostic validation, clinicians ask additional questions to validate existing symptoms from an external perspective through their clinical expertise. The detailed process of the Klenico system is illustrated in Figure 1. The combination of self-report and external assessment is critical for a standardized diagnostic procedure, and this multimodal approach is widely considered the ideal diagnostic method. Therefore, the modular structure of Klenico can facilitate efficient and comprehensive diagnostic procedures that can be easily integrated into everyday clinical practice while still adhering to a standardized diagnostic approach.

Klenico has been successfully used in clinical practice. However, to date, the psychometric quality criteria have not yet been formally analyzed. Therefore, this study aimed to take a first step toward validating the Klenico system. The focus was to ascertain the criterion validity of the self-report module by comparing it against a diagnostic gold standard. In addition, the module’s overlap with other standard self-report questionnaires was investigated to examine its convergent and divergent validity.

## Methods

### Procedure

This study used fully anonymized data from routine diagnostic procedures at a psychotherapeutic outpatient clinic for teaching and research conducted between October 2018 and May 2021. The outpatient clinic offers evidence-based psychotherapy for children, adolescents, and adults with mental-health problems. Upon admission, adult patients were assigned to routine diagnostic procedures, which consisted of a multi-step assessment. First, patients were given access to the Klenico self-report module by the clinic staff and they completed it on a device of their choosing (computer, mobile phone, or tablet) individually at home. In the next step, patients participated in an initial consultation, followed by a diagnostic interview with the SCID. Finally, patients completed a series of self-report questionnaires at home. The interviewers were licensed psychotherapists or psychotherapists trained under supervision. We obtained written informed consent to use anonymized data from all patients.

### Measures

**Structured Clinical Interview for DSM-IV (SCID).** The German version of the SCID (Wittchen et al., 1997) was used to establish the ICD-10 diagnoses. The SCID is a semi-structured diagnostic interview based on DSM-IV criteria (Sass et al., 1996). It is a frequently used and well-established clinical interview and is considered a diagnostic gold standard (Steinlechner et al., 2015).

**Beck Depression Inventory (BDI-II)**. The BDI-II assesses the severity of depressive symptoms (Beck et al., 1996). The questionnaire contains 21 items based on the DSM-IV criteria for major depression (Kühner et al., 2007). Each statement was rated on a scale from 0 to 3, with 0 indicating no difficulty and 3 indicating the highest severity of difficulty. All items were summed up with a possible total score of 63. The BDI-II is a well-established questionnaire that has demonstrated criterion validity with the SCID-I, high associations with other depression questionnaires, and a test-retest reliability between 0.73 and 0.96 (Wang & Gorenstein, 2013). Cronbach’s alpha was 0.93 in the current study.

**Brief-Symptom-Checklist (BSCL)**. The BSCL assesses impairment on nine different symptom dimensions based on 53 items (somatization, OCD, interpersonal sensitivity, anxiety, depression, aggressivity/hostility, phobic anxiety, paranoid ideation, and psychoticism). Items were rated on a 4-point Likert scale ranging from 0 (not at all) to 4 (very strong). The BSCL has satisfactory test-retest reliability (Franke, 2017) and good convergent and divergent validity but insufficient factorial validity (Bolbeth et al., 2021). The items were summed up separately for each scale. In the current study, the Cronbach’s alpha values ranged from 0.68 for the psychoticism scale to 0.87 for the depression scale.

**Patient Health Questionnaire (PHQ-D)**. The PHQ-D was developed for the screening of mental disorders based on the DSM-IV and consists of 78 items. It demonstrates good construct validity and a sensitivity and specificity of 85% and 70%, respectively, compared to SCID-I diagnoses (Gräfe et al., 2004). The PHQ scales for GAD and somatoform disorders were employed in the diagnostic procedures, from which sum scores were calculated. Cronbach’s alpha for PHQ somatization and PHQ GAD were 0.79 and 0.86, respectively.

**Klenico Self-Report Module.** The detailed procedure of the self-report module is illustrated in Figure 1. On average, 232 items were presented in the detailed assessment to the patients. Only the numerical values (ranging from 0 to 100) from the self-report module are used in this study. Mean scores for each Klenico domain scale and separate scores for particular disorder scales were calculated.

### Sample

Data from 115 adult patients were used (n = 65 female, n = 49 male, n = 1 unspecified). The patients had a mean age of 36.4 years (SD = 13.3, range = 19–72). Table 1 presents the ICD-10 diagnostic group frequencies (for a full overview, see Table S1 in the supplementary material). Approximately 60% of the surveyed patients received only one diagnosis, whereas the others had comorbidities with at least one other mental disorder.

**Table 1.** Frequencies of the relevant analyzed ICD-10 diagnostic categories. Groups were formed by assigning each individual diagnosis of each patient to the corresponding disorder domain. Multiple diagnoses are possible. Listed frequencies are relative to the total number of given diagnoses.

Diagnostic group	ICD-10 codes	Frequency	%
Depressive disorders	F32.x-34.x	56	31.8
Anxiety disorders	F40.xx-41.x	25	14.2
Eating disorders	F50.x	24	13.6
Reaction to severe stress, and adjustment disorders	F43.x	15	8.5
Somatoform disorders	F45.xx	9	5.1
OCD	F42.x	7	4.0
Behavioral and emotional disorders with onset usually occurring in childhood and adolescence	F9x.x	6	3.4
Schizophrenia	F20.0	2	1.1

### Statistical analyses

All data analyses were performed using the RStudio software (Version 2021.9.1.372, Boston, MA, USA). *P-*values less than 0.05 were considered significant.

The internal consistency of the analyzed Klenico domain/disorder scales and routine questionnaire scales was measured using Cronbach’s alpha.

To evaluate the criterion validity for each Klenico domain scale, group differences between the means of patients with the corresponding diagnosis and other diagnoses were compared using one-way analysis of variance (ANOVA). Only diagnostic groups with at least four patients were included in the analysis. The following Klenico domain scales were considered: anxiety disorders, depressive disorders, somatoform disorders, OCD, stress-associated disorders, eating disorders, and ADHD

Convergent and divergent validity were tested by calculating Pearson’s correlation coefficients between the Klenico domain/disorder scales and corresponding or diverging self-report questionnaire scales. The following Klenico domain/disorder scales were included in the analyses: anxiety disorders (panic disorder, agoraphobia, social phobias, specific phobias, and GAD), depressive disorders, eating disorders (anorexia, bulimia), psychotic disorders, somatoform disorders, OCD, ADHD, and stress-associated disorders. Following standard conventions, correlation coefficients of 0.10–0.29, 0.30–0.49, and ≥ 0.50 were considered small, medium, and large, respectively (Cohen, 2013).

### Ethics statement

The current study used retrospectively fully anonymized data. Therefore, it does not fall under the Human Research Act of Switzerland, and no formal ethical approval is required for this type of study.

## Results

### Internal consistency

All analyzed Klenico scales reached Cronbach’s alpha coefficients of 0.8 or higher, except for the psychostic disorders domain scale, which showed a value of 0.62. For further details, refer to Table S2.

### Criterion validity

Patients with particular SCID-diagnoses demonstrated significantly higher mean scores in the respective Klenico domain scales for anxiety disorders, depressive disorders, OCD, stress-associated disorders, ADHD, and eating disorders compared to patients with non-corresponding diagnoses. The only scale with no significant differences was that for somatoform disorders. However, there was a trend toward a higher mean score for the corresponding diagnosis (see Table 2).

**Table 2.** Diagnostic group differences resulting from the one-way ANOVA. ADHD = attention deficit hyperactivity disorder; OCD = obsessive-compulsive disorder; SD = standard deviation.

	Corresponding ICD-10 diagnosis			Other ICD-10 diagnosis				
Klenico domains	*Mean*	*SD*	*N*	*Mean*	*SD*	*N*	F-value	*p*-value
Anxiety disorders	18.03	11.31	25	9.27	9.98	90	14.21	<0.001
Depressive disorders	26.61	17.43	56	15.63	17.08	59	11.65	<0.001
Somatoform disorders	9.70	5.35	9	5.36	8.15	106	2.45	0.120
OCD	26.14	10.54	7	7.10	8.68	108	30.80	<0.001
Stress-associated disorders	17.51	15.91	15	7.42	7.94	100	15.32	<0.001
Eating disorders	34.97	20.12	24	7.70	12.96	91	65.40	<0.001
ADHD	17.03	14.56	4	7.10	8.39	111	5.13	0.025

### Construct validity

The Klenico GAD scale was highly associated with the equivalent BSCL anxiety scale, while the other Klenico anxiety scales revealed moderate associations. Moreover, all the Klenico anxiety scales revealed significant associations with the BSCL phobic anxiety scale, with the corresponding panic disorder and agoraphobia scales correlating in the high range. In addition, while there was a significant association between all Klenico anxiety scales, except for the specific phobias scale and the PHQ GAD scale, the corresponding Klenico GAD scale revealed the highest correlation coefficient. The BSCL interpersonal sensitivity scale showed large correlations with the corresponding Klenico social phobias scale, followed by the GAD and agoraphobia scales, but no association with the Klenico panic disorder and specific phobias scales (see Table 3).

**Table 3.** Pearsons’ correlation coefficients between Klenico anxiety disorders scales and convergent self-report questionnaire scales. GAD = Generalized anxiety disorder. *** *p* < 0.001; ** *p* < 0.01; * *p* < 0.05.

	Corresponding self-report scales			
Klenico anxiety disorders	BSCL anxiety	BSCL phobic anxiety	PHQ GAD	BSCL interpersonal sensitivity
Panic disorder	0.45***	0.51***	0.23*	0.07
Agoraphobia	0.31**	0.55***	0.24*	0.38***
Social phobias	0.31**	0.39***	0.23*	0.65***
Specific phobias	0.32**	0.42***	0.12	0.19
GAD	0.63***	0.48***	0.46***	0.52***

For the Klenico depressive disorders domain scale, very high correlations were apparent for both the BDI-II (r = 0.70, *p* < 0.001) and the BSCL depression (r = 0.69, *p* < 0.001) scale. Likewise, there was a strong correlation between the Klenico somatoform disorders domain scale and both the BSCL somatization (r = 0.51, *p* < 0.001) and the PHQ somatization (r = 0.51, *p* < 0.001) scales. A similar pattern was found for Klenico psychotic disorders domain scale and BSCL paranoid ideation (r = 0.39, *p* < 0.001), as well as the BSCL psychoticism (r = 0.31, *p* = 0.006) scale. Similarly, the Klenico OCD scale was correlated with the BSCL OCD scale (r = 0.33, *p* = 0.003).

Table 4 reports the correlations between the Klenico anxiety disorder scales and divergent self-report scales. There were significant associations above r = 0.40 between Klenico agoraphobia, social phobias, and GAD with both the BSCL depression and BDI-II as well as BSCL psychoticism and paranoid ideation scales. Similarly, the Klenico GAD and panic disorder scales were correlated with the BSCL and PHQ somatization scales. Additionally, the Klenico GAD scale was correlated the BSCL OCD scale. All other associations were either non-significant or within a small range (below r = 0.40).

**Table 4.** Pearson correlation coefficients for divergent validity regarding Klenico anxiety disorders. GAD = Generalized anxiety disorder. *** *p* < 0.001; ** *p* < 0.01; * *p* < 0.05.

	Divergent self-report questionnaires							
Klenico anxiety disorders	BDI-II	BSCL depression	BSCL paranoid ideation	BSCL psychoticism	BSCL somatization	PHQ somatization	BSCL OCD	BSCL hostility
Panic disorder	0.30**	0.10	0.18	0.18	0.45***	0.42***	0.20	0.17
Agoraphobia	0.44***	0.38***	0.38***	0.42***	0.26*	0.35**	0.32**	0.18
Social phobias	0.45***	0.44***	0.45***	0.43***	0.22*	0.35**	0.38***	0.30*
Specific phobias	0.14	0.13	0.33**	0.22	0.34**	0.28*	0.15	0.21
GAD	0.56***	0.45***	0.56***	0.49***	0.42***	0.48***	0.52***	0.37***

The Klenico depressive disorders domain scale was correlated with all other self-report scales in the moderate to high range, except for the BSCL somatization scale. Regarding the Klenico psychotic disorders domain scale, moderate associations were apparent with the BSCL phobic anxiety and BSCL depression scales. In addition, the Klenico somatoform disorders domain scale and OCD scales correlated with the BSCL anxiety scale in the moderate range.

Finally, the Klenico stress-associated disorder domain scale showed moderate-to-large correlations with the BSCL depression scale and BDI-II, BSCL psychoticism, and BSCL (phobic) anxiety. All other associations with the self-report questionnaires were either non-significant or below r = 0.40. Further details are provided in Table 5.

**Table 5.** Pearson correlation coefficients for divergent validity regarding other Klenico domains and disorders. *** *p* < 0.001; ** *p* < 0.01; * *p* < 0.05. ADHD = attention deficit hyperactivity disorder; OCD = obsessive-compulsive disorder; Interp. sens. =  Interpersonal sensitivity; Dep. =  Depression; Par. id. =  Paranoid ideation; Psych. =  Psychoticism; Soma. =  Somatization; Host. =  Hostility. NA = not applicable as included in convergent validity.

	Divergent self-report questionnaires											
Klenico domains/ disorders	BSCL anxiety	BSCL phobic anxiety	PHQ GAD	BSCL interp. sens.	BDI-II	BSCL dep.	BSCL par. id.	BSCL psych.	BSCL soma.	PHQ soma.	BSCL OCD	BSCL host.
Depressive disorders	0.51***	0.58***	0.41***	0.63***	*NA*	*NA*	0.61***	0.60***	0.30*	0.47***	0.59***	0.45***
Psychotic disorders	0.25	0.40***	0.22*	0.34***	0.37***	0.41***	*NA*	*NA*	0.13	0.23*	0.37**	0.22*
Somatoform disorders	0.42***	0.34*	0.24*	0.14	0.17	0.09	0.33**	0.14	*NA*	*NA*	0.31*	−0.06
OCD	0.45***	0.38**	0.21*	0.24*	0.30**	0.13	0.23*	0.24*	0.28**	0.20	*NA*	0.28*
Anorexia	0.17	0.19	0.18	0.37**	0.36***	0.31**	0.32**	0.34**	0.13	0.33**	0.06	0.26*
Bulimia	0.22	0.20	0.16	0.34**	0.24*	0.30**	0.36**	0.26*	0.21	0.32**	0.12	0.35**
ADHD	0.02	0.09	−0.07	−0.01	0.06	−0.09	0.06	−0.11	−0.06	−0.07	0.22	−0.13
Stress-associated disorders	0.40***	0.48***	0.12	0.31**	0.52***	0.40***	0.37***	0.46***	0.35**	0.39***	0.33*	0.37**

## Discussion

Klenico was developed to increase diagnostic accuracy by approaching the diagnostic state-of-the-art procedures. This study sought to take a first step towards validation by focusing on the self-report module.

For criterion validity, patients with a corresponding diagnosis had significantly higher mean scores than those with other diagnoses. A non-significant trend was observed only for the somatoform disorders domain. Most likely, a very low prevalence (5%) in addition to high comorbidity (65%) in this specific sample can account for this finding. Thus, the results indicated that the Klenico self-report module can reliably differentiate between different mental disorders.

Regarding convergent validity, as expected, moderate-to-large correlations were found with the corresponding questionnaires. Associations were highest for scales reflecting similar constructs of the underlying disorder conceptualization. However, while still significant, converging correlations between paranoid/psychotic scales and OCD scales were only in the moderate range. This finding might be explained by the conceptualization of the self-report questionnaires used for the comparison. The BSCL was constructed to assess symptom dimensions; accordingly, these scales reveal substantial heterogeneity with respect to the underlying disorder (Newson et al., 2020). In particular, the paranoid ideation and psychoticism scales have been criticized for presenting a highly heterogeneous construct (Pedersen et al., 2016). The same applies to the BSCL OCD scale. Previous studies have suggested that items are too non-specific and include depressive constructs (Tritt et al., 2010).

Regarding divergent validity, the associations with most questionnaires were non-significant or low. However, there were exceptions with moderate to high correlations, particularly with the BSCL scales. While this finding might seem striking at first glance, it could be explained by the construction of the BSCL as a non-disorder-specific instrument, which therefore covers the features of several mental disorders on the same scale (Franke, 2017). In addition, these associations might reflect heterogeneity in ICD-10 disorder conceptualization. For instance, depressive disorders are often associated with increased feelings of anger (Dutton & Karakanta, 2013) and patients can present somatic features (Haug et al., 2004). A similar explanation applies to psychotic symptoms, which can be a core feature of severe depressive disorders. Moreover, depression can involve negative expectations for the future (Miranda & Mennin, 2007), which is similar to the items outlined in the PHQ GAD scale. Similarly, a broad spectrum of psychotic disorders includes symptoms of anxiety and affective disturbances (Buckley et al., 2009), and stress-related disorders often manifest themselves in associated symptoms of altered mood and cognition (Sareen, 2014). Finally, the Klenico somatoform disorder scale mirrors the ICD-10 concept, which includes hypochondria. Accordingly, an overlap between this Klenico domain and the BSCL anxiety scale is not surprising. Taken together, these findings do not compromise the divergent validity of the Klenico system but are likely to reflect the weaknesses of the respective scales used for the comparison.

Overall, the results concerning the criteria and construct validity provide the first evidence that the Klenico self-report module is a valid diagnostic tool. Thus, these preliminary results highlight the potential usefulness of the Klenico system as a time-efficient and standardized diagnostic tool that may facilitate early and accurate diagnoses. This is particularly important because a correct and timely diagnosis is crucial for successful treatment outcomes (Bukh et al., 2013; Cheung et al., 2017). A strength of this study is that the Klenico self-report module was tested in a routine clinical setting characterized by patients with high levels of comorbidity, thus reflecting high ecological validity. Therefore, because of the broad age range of the sample, it can be assumed that the results can be generalized to psychotherapeutic outpatient and comparable settings. However, severely affected patients may be less likely in these setting and people with a higher socio-economic status are probably more likely to use outpatient services (Klein & von dem Knesebeck, 2016). Therefore, it is possible that generalizability to the general clinical population is limited to some extent.

In addition, the self-report module was compared to SCID, which is considered the gold standard for diagnosis (Nakash et al., 2015). The fact that the Klenico self-report module can reliably differentiate between SCID-based ICD-10 diagnoses further underlines its potential.

### Future outlook

In this work, regular, established methods were used to validate the Klenico system, namely, cross-validation with other clinical measurements, validated questionnaires, and interviews. Although this procedure is generally highly accepted and meaningful in the field of psychology, it may lack explanatory power that goes beyond these clinical measurements as it considers only methods within the same framework (Zachar et al., 2015). This reflects a general issue in psychological diagnostics, namely that mental disorders represent theoretical constructs consisting of a specific set of symptoms. While these are useful for classification in the existing framework, their underlying etiology has not yet been determined, and their neurobiological equivalents are still missing (Jablensky, 2010). This is also demonstrated by the fact that heterogeneous constructs may be used for different questionnaires, which can lead to limitations in comparability, such as the BSCL used in this study.

Therefore, it may be useful to validate psychological diagnostic tools translationally with neurobiological equivalent measurements such as fMRI approaches. This approach offers the potential to gain additional evidence of validity, to make mental disorders neurobiologically comprehensible, and to link the underlying neurobiological processes to psychological questionnaires (Stoyanov, 2014). Initial findings have already been obtained by validating psychological questionnaires with fMRI measurements (Stoyanov et al., 2018). However, the methodology still needs to be further developed in terms of validity and the linkage between neuroimaging and psychological diagnostic tools (Stoyanov et al., 2012; Zachar et al., 2015). Therefore, it seems promising to validate the Klenico system using neuroscientific measurement methods to provide external validity and link the system to underlying etiologies.

### Limitations

This study has several limitations. First, the sample size is small. Consequently, the patients had to be summarized in the ICD-10 diagnostic groups, thus compromising diagnostic homogeneity. In addition, there were few patients diagnosed with ADHD, OCD, and somatoform disorders. Therefore, these results should be considered preliminary and require further validation. Second, not all disorder domains present in the Klenico system could be investigated since either prevalence rates were too low in the sample or converging self-report measures for these domains were not included in routine assessments at the outpatient clinic. In particular, the Klenico dementia, behavioral disorders due to psychoactive substances, mania, autism spectrum disorders, and personality disorder domains were not considered. The same applies to specific disorders within one Klenico domain that cannot be analyzed separately, except for eating and anxiety disorders.

Finally, only the self-report module was completed and evaluated in the current study. As such, the diagnostic validation module could not be tested at this stage.

## Conclusion

In summary, the analysis supports the validity of the Klenico self-report module. Future research should further examine larger samples to allow for more fine-grained analyses and additional indices of validity, such as structural models. Moreover, the domains that were not considered in this study need to be addressed. In addition, further clarification with more disorder-specific questionnaires is needed, particularly tools that have less heterogeneity in the underlying constructs than the currently used BSCL. Finally, further investigation of the clinical validation module of the Klenico system is needed to generate initial evidence for its psychometric properties.

The current study presents a meaningful first step in establishing the psychometric properties of the overall Klenico system. Therefore, it might be an effective tool for accurate diagnostic procedures in outpatient settings and could help to efficiently close the gap in the current practice of unstandardized diagnostic procedures. This can provide benefits for both patients and the healthcare system.

## Author note

Stefan Reutimann, Klenico Health AG, University of Zurich Startup; David Milanovic, Klenico Health AG, University of Zurich Startup; Marco D. Gulewitsch, Department of Psychology, Faculty of Science, University of Tübingen; Mareike Augsburger, Klenico Health AG, University of Zurich Startup.

## Supplementary Material

Supplemental Material

## Data Availability

The data and materials of this study, including the R code, are available upon request from the authors.
