# Supplementary material for: Preliminary validation of the Klenico diagnostic software self-report module through comparison with the diagnostic gold standard in an outpatient routine clinical sample
Source: Health Psychol Behav Med. 2023 Aug 30;11(1):2244576. doi: 10.1080/21642850.2023.2244576 (PMC10469457; doi:10.1080/21642850.2023.2244576)
Supplement: Supplemental Material [file RHPB_A_2244576_SM8519.docx]

**Electronic Supplementary material**

| **Diagnostic group** | **ICD-10 codes** | **Frequency** | **%** |
| --- | --- | --- | --- |
| Depressive disorders | F32.x-34.x | 56 | 31.8 |
| Anxiety disorders | F40.xx-41.x | 25 | 14.2 |
| Eating disorders | F50.x | 24 | 13.6 |
| Disorders of adult personality and behaviour | F6x.xx | 17 | 9.7 |
| Reaction to severe stress, and adjustment disorders | F43.x | 15 | 8.5 |
| Somatoform disorders | F45.xx | 9 | 5.1 |
| Obsessive-compulsive disorder | F42.x | 7 | 4.0 |
| Behavioural and emotional disorders with onset usually occurring in childhood and adolescence | F9x.x | 6 | 3.4 |
| Mental and behavioural disorders due to psychoactive substance use | F1x.x | 6 | 3.4 |
| Bipolar affective disorders | F31.xx | 5 | 2.8 |
| Other Behavioural syndromes associated with physiological disturbances and physical factors | F5x.x | 4 | 2.3 |
| Schizophrenia | F20.0 | 2 | 1.1 |

Table S1: Frequencies of ICD-10 diagnostic categories. Groups were formed by assigning each individual diagnosis of each patient to the corresponding disorder domain. Multiple diagnoses are possible. Listed frequencies are relative to the number of given diagnosis.

| **Klenico domains/disorders** | **Cronbach’s alpha** | **Number of items** | **Mean** | **SD** |
| --- | --- | --- | --- | --- |
| Anxiety disorders | 0.96 | 88 | 11.18 | 10.85 |
| Panic disorder | 0.95 | 20 | 9.94 | 15.74 |
| Agoraphobia | 0.92 | 22 | 6.27 | 10.83 |
| Social anxiety | 0.89 | 15 | 15.39 | 16.49 |
| Specific phobia | 0.82 | 15 | 6.76 | 9.64 |
| GAD | 0.89 | 12 | 22.12 | 21.16 |
| Depressive disorders | 0.94 | 34 | 20.98 | 18.04 |
| Psychotic disorders | 0.62 | 13 | 5.48 | 6.56 |
| Somatoform disorders | 0.94 | 63 | 5.70 | 8.04 |
| OCD | 0.87 | 23 | 8.26 | 9.87 |
| Eating disorders | 0.95 | 26 | 13.39 | 18.39 |
| Anorexia nervosa | 0.87 | 13 | 11.67 | 14.94 |
| Bulimia nervosa | 0.97 | 12 | 15.25 | 25.69 |
| ADHD | 0.93 | 31 | 6.91 | 10.42 |
| Stress-associated disorders | 0.90 | 35 | 8.74 | 9.88 |

Table S2: Internal consistencies of the tested Klenico domains/disorders.
